# Klippel–Trenaunay–Weber syndrome with atypical presentation of hypersplenism and nephrotic syndrome: a case report

**DOI:** 10.1186/s13256-017-1413-1

**Published:** 2017-08-21

**Authors:** Linda Kundzina, Sandra Lejniece

**Affiliations:** 1 Faculty of Medicine, Riga Stradins University, Riga, Latvia; 2 Department of Internal Diseases, Riga Stradins University, Riga, Latvia; 3 Riga East Clinical University Hospital, Chemotherapy and Hematology Clinic, Riga, Latvia

**Keywords:** Klippel–Trenaunay–Weber syndrome, Hypersplenism, Nephrotic syndrome

## Abstract

**Background:**

Klippel–Trenaunay–Weber syndrome is a rare syndrome; unfortunately, very few studies of the connection between hypersplenism, nephrotic syndrome, and Klippel–Trenaunay–Weber syndrome have been published.

**Case presentation:**

We report the case of a 40-year-old white man with a typical clinical presentation of Klippel–Trenaunay–Weber syndrome, including “port-wine stains,” varicose veins, hypertrophy of lower extremities, and arteriovenous fistula, as well as an unfortunate development of hypersplenism and nephrotic syndrome.

**Conclusions:**

This case report described considerable atypical relevance of Klippel–Trenaunay–Weber syndrome and hypersplenism together with nephrotic syndrome. A multidisciplinary approach was made. Unfortunately, hypersplenism is characterized by pancytopenia that suggests splenectomy, whereas nephrotic syndrome is an indication for renal biopsy; the splenectomy and renal biopsy were delayed due to our patient’s severe condition. Deeper analysis including study of other patients with Klippel–Trenaunay–Weber syndrome would help us to understand the connection between elevated spleen and liver sizes, nephrotic syndrome, and Klippel–Trenaunay–Weber syndrome.

## Background

Klippel–Trenaunay–Weber syndrome (KTWS), defined as a sporadic disorder, is characterized by “port-wine stains,” lymphatic anomalies, and varicose veins in association with variable overgrowth of soft tissue and bone that is present at birth together with arteriovenous malformations [1–3]. The diagnosis of KTWS is based on physical signs and symptoms, as well as genetic testing; computed tomography (CT), magnetic resonance imaging (MRI), and Doppler studies may be useful in determining the extent of the condition and the best way for managing it [2–5]. Very few studies of a connection between hypersplenism, nephrotic syndrome, and KTWS have been published.

## Case presentation

**Fig. 1:**
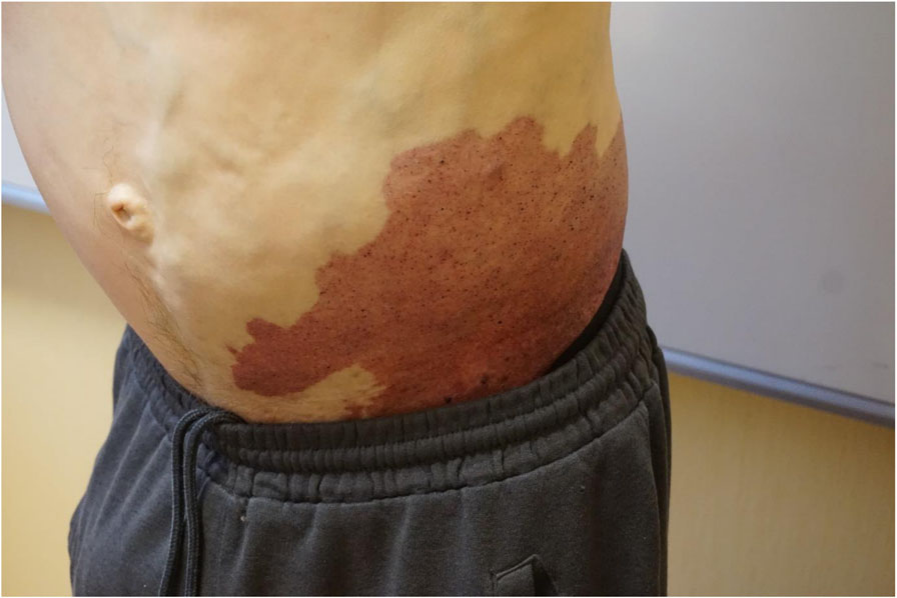
Port-wine stains

**Fig. 2:**
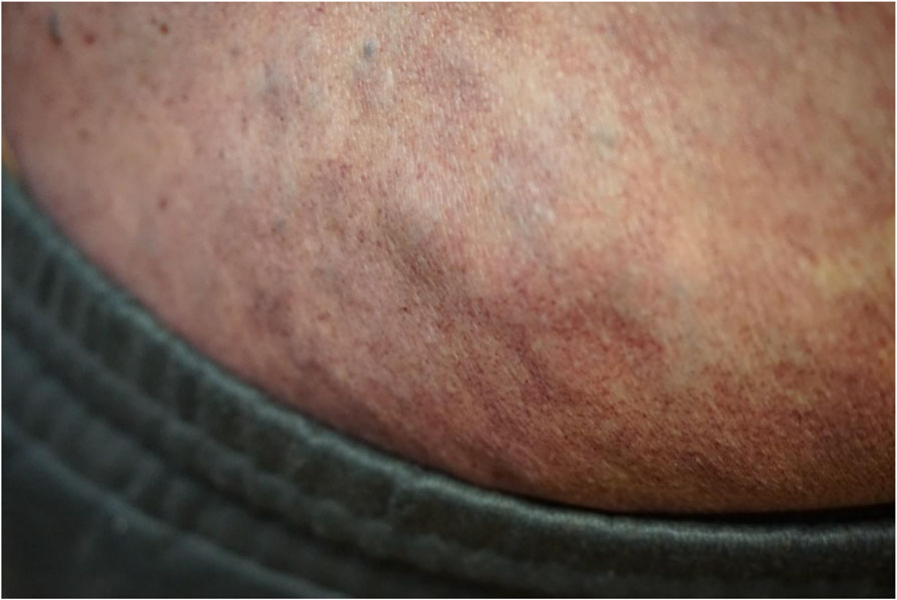
Port-wine stains

**Fig. 3:**
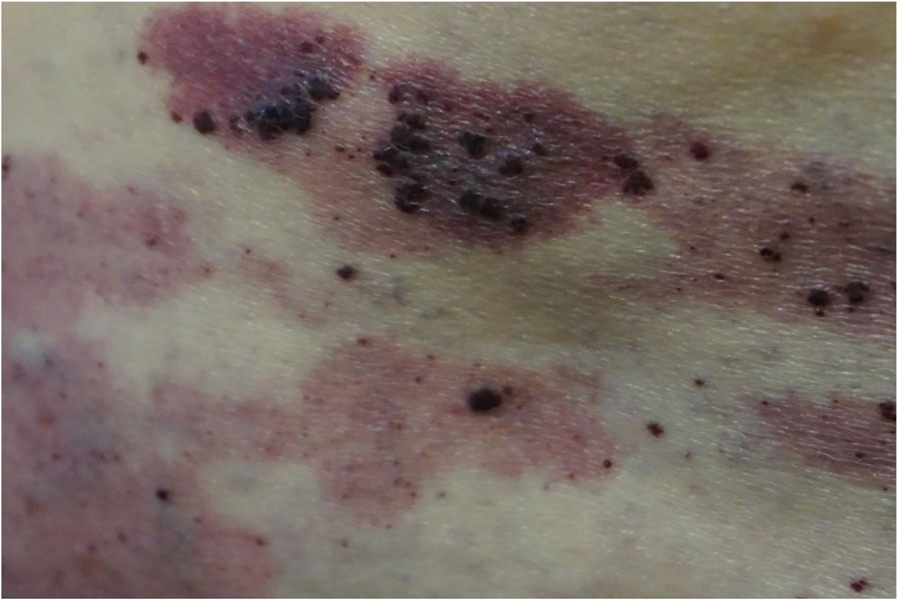
Port-wine stains

**Fig. 4:**
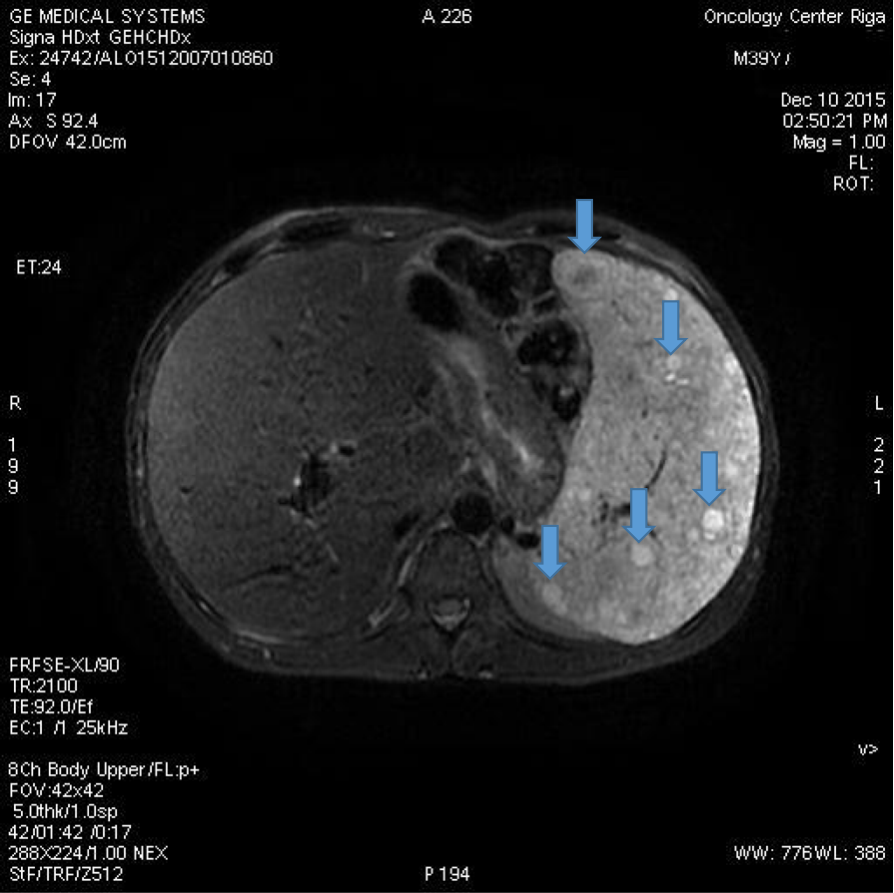
Magnetic resonance imaging (MRI) showing hepatosplenomegaly with multiple hemangiomas in spleen (*arrows*)

**Fig. 5:**
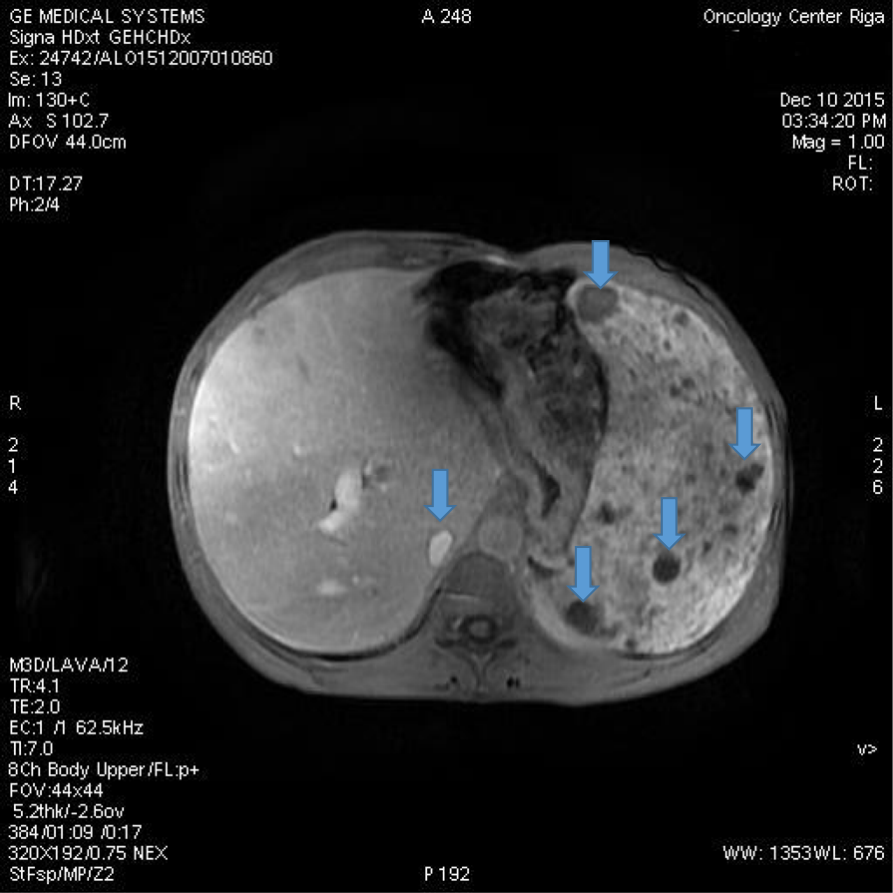
Magnetic resonance imaging (MRI) showing hepatosplenomegaly with multiple hemangiomas in spleen and liver (*arrows*)

**Fig. 6:**
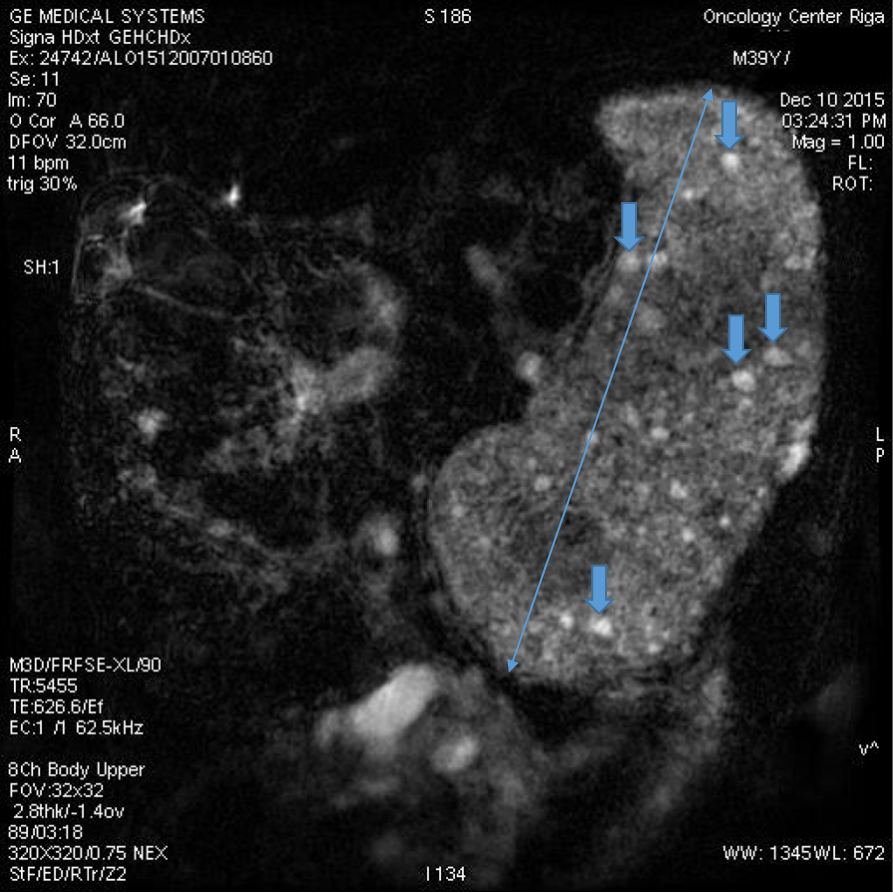
Magnetic resonance imaging (MRI) showing splenomegaly with multiple hemangiomas (*arrows*)

A 40-year-old white man with a disease history of KTWS diagnosed since birth presented with iron deficiency anemia, his hemoglobin (Hb) level was 9 g/dL, and complaints about episodes of hematuria and weakness. The remarkable findings of a physical examination were port-wine stains and varicose veins on his limbs and part of his torso (Figs. 1, 2, and 3), and hypertrophy of both lower extremities: status after amputation of the digits of his left foot and soft tissue resection of plantar surface with auto-skin plastic; and arteriovenous fistula in both legs. MRI and Doppler studies showed: splenomegaly (size 20 × 12 cm) since 1998; multiple hemangiomas in spleen, liver, and bladder; hepatomegaly since 2009 (Figs. 4, 5 and 6); and varicose mesenteric veins. A CT scan in 1998 revealed phleboliths in his pelvis around his colon and bladder. Dynamic renal scintigraphy in 2016 showed slowed excretion due to minor disorder of vascularization of both kidneys with weakened renal function. Laboratory results displayed pancytopenia consisting of normochromic normocytic anemia (Hb, 9 g/dL), neutropenia (1.24 × 103/uL) and thrombocytopenia (107 × 103/uL). Biochemical tests revealed low 25-hydroxyvitamin D (10.67, normal range 30 to 100 ng/ml), fibrinogen (1.1, normal range 1.8 to 3.6 g/l), calcium (1.95, normal range 2.08 to 2.65 mmol/l), and normal creatinine and parathyroid hormone (PTH) level. Hypoalbuminemia (27, normal range 35 to 52 g/l), hyperlipidemia (2.31, normal range < 1.7 mmol/l), and proteinuria (6.85 g/L) described the main criteria for nephrotic syndrome. During hospitalization he received multiple transfusions of red blood cells, perindopril, amlodipine, atorvastatin, allopurinol, and Vigantoel (vitamin D_3_).

## Discussion

This case showed a typical presentation of KTWS, including signs like port-wine stains, varicose veins, hypertrophy of low extremities, and arteriovenous fistula. However, an unusual presentation of KTWS was also displayed: KTWS and nephrotic syndrome combined with hypersplenism, which was probably caused by numerous hemangiomas found in his spleen and liver. Splenectomy was suggested due to multiple transfusions of red blood cells, which were prescribed during his hospitalization and did not lead to long-term improvement. A renal biopsy, which is performed to verify cause of nephrotic syndrome, was delayed due to his severe condition. The connection between KTWS, hypersplenism, and nephrotic syndrome is still unclear due to the rare presentation of KTWS itself. A renal biopsy could help to identify the cause of nephrotic syndrome, leading to a better comprehension of the unclear connection between KTWS, hypersplenism, and nephrotic syndrome.

## Conclusions

Signs like port-wine stains, varicose veins, hypertrophy of lower extremities, and arteriovenous fistula are typical of KTWS. This case showed considerable atypical relevance: KTWS and hypersplenism together with nephrotic syndrome. Unfortunately, hypersplenism is characterized by pancytopenia that suggests splenectomy, whereas nephrotic syndrome is an indication for renal biopsy; the splenectomy and renal biopsy were delayed due to our patient’s severe condition. Deeper analysis including study of other patients with KTWS would help us to understand the connection between elevated spleen and liver sizes, nephrotic syndrome, and KTWS.
